# Exploring the therapeutic potential of plasma from intermittent fasting and untreated rats on aging‐induced liver damage

**DOI:** 10.1111/jcmm.18456

**Published:** 2024-06-25

**Authors:** Hüseyin Allahverdi

**Affiliations:** ^1^ Department of Molecular Biology and Genetics Muş Alparslan University Muş Turkey

**Keywords:** inflammation, intermittent fasting, liver, Notch signalling, plasma exchange

## Abstract

This research aims to investigate the effects of plasma from 12‐month‐old intermittently fasting rats (IFpls) and untreated rats (Npls) on the liver biomolecules and histological changes in 24‐month‐old male Sprague–Dawley rats. Fasting rats underwent an 18‐h daily fasting period and a 6‐h feeding window for 35 days. The plasma was administered bi‐daily, and blood samples were examined for specific liver biomolecules. Fourier transform infrared (FTIR) spectroscopy and linear discriminant analysis (LDA) was used to identify molecular profiles. Liver sections were stained for histopathological evaluation, and the expression levels of Notch signalling pathway components were assessed. Distinct molecular profiles were identified across liver biomolecules, lipids, proteins and nucleic acids with high accuracy. Notably, IFpls was found to protect against hepatic instability, microvesicular steatosis and liver fibrosis by decreasing lymphatic infiltration density and Notch pathway expression levels. Both treatments reduced protein oxidation and carbonylation, with Npls showing a pronounced decrease in protein oxidation. Furthermore, Npls increased protein conformation and glycogen/phosphate content, while IFpls increased glucose/protein content. Both IFpls and Npls induce substantial and unique alterations in liver biomolecules. IFpls offers a protective effect on various liver conditions, while Npls exhibits promising results in reducing protein oxidation and altering biomolecule content. These findings offer valuable insights for future research and potential therapeutic approaches.

## 1 INTRODUCTION

Aging is a universal biological process that involves a progressive decline in physiological functions, which predisposes individuals to various chronic diseases, including liver disorders. The liver, as a central organ involved in metabolic, detoxification and synthetic processes, is particularly susceptible to age‐related deterioration, leading to fibrosis, steatosis and reduced regenerative capacity.^1^ This decline in liver function associated with aging is exacerbated by oxidative stress and chronic low‐grade inflammation, also known as ‘inflammaging’.^2^

Recent research has demonstrated that intermittent fasting (IF), a pattern of eating that involves periods of voluntary abstinence from food and caloric beverages, has significant effects on health and longevity.^3^, ^4^ Studies suggest that IF enhances autophagy, improves metabolic profiles and reduces oxidative stress and inflammation in various tissues, including the liver.^5^ The protective and rejuvenative effects of IF may, in part, be mediated through alterations in the composition and functionality of circulating biomolecules, such as increased levels of sirtuins and reduced incidence of protein carbonylation, thus promoting cellular health and stability.^6^ Recent evidence also suggests that IF may also promote rejuvenation by increasing the generation of new cells in various tissues, such as the ileum, colon and liver.^7^

Furthermore, the therapeutic potential of plasma exchange with young or conditionally‐altered plasma, such as from fasting individuals, presents a novel avenue for mitigating age‐related ailments.^8^ Young plasma is rich in growth factors and other rejuvenative proteins that can stimulate tissue repair and counteract aging‐related cellular damage.^9^ Clinical and preclinical studies have demonstrated that transfusions of young plasma can enhance cognitive function, improve physical performance and reduce markers of systemic inflammation.^10^ Additionally, young plasma has been found to improve cardiovascular function, reduce inflammation and bolster the immune system.^11^ In an effort to further explore the potential of young plasma transfusions, recent studies have investigated their effects on aged gut microbiota, demonstrating an increase in alpha diversity following transfusions.^12^, ^13^ Moreover, another study has highlighted the therapeutic potential of young plasma in mitigating age‐related changes and inflammation in the intestinal tract. The study revealed rejuvenating effects on aged ileum and colon tissues, suggesting a therapeutic potential for intestinal aging.^14^

In this regard, this study examines the effects of plasma from 12‐month‐old rats subjected to intermittent fasting (IFpls) and untreated control rats (Npls) on the liver biomolecules and histological structure in 24‐month‐old male Sprague–Dawley rats. This investigation seeks to unravel whether IF‐induced alterations in plasma composition can attenuate liver damage associated with aging more effectively than plasma from non‐fasted young rats. We hypothesize that IFpls confers a protective effect against hepatic instability, microvesicular steatosis and liver fibrosis, potentially offering a therapeutic strategy to enhance liver health in the elderly. Through this research, we aim to contribute novel insights into the mechanisms by which dietary interventions such as intermittent fasting could modulate the biochemical landscape of aging.

## 2 MATERIALS AND METHODS

### 2.1 Animal studies

Intermittent fasting was applied to the rats (12‐month old, *n* = 17) in the study's for 35 days. While the rats in the fasting group were always able to drink water, their access to food was restricted for 18 h, and they were only allowed to feed for 6 h. The food access interval of the animals in the experimental group was determined to be between 09:00 AM and 03:00 PM. At the end of the intermittent fasting application, the plasma of the rats (*n* = 17) who underwent intermittent fasting for 35 days and the rats at the same age without any application (*n* = 17) were taken and sacrificed. Plasma taken from rats administered IF was transferred to one group (*n* = 7), and plasma from rats without any treatment was transferred to 24 month old Sprague Dawley rats in another group (*n* = 7) once every 2 days, 0.5 mL to each rat 15 times. No application was made to the rats in the control group (*n* = 7). All animals fed ad libitum with a standard rodent diet and housed under standard animal care. All animals were ether‐stunned and sacrificed the day after. Liver tissues were collected, immediately shocked on dry ice, and stored at −80°C until analysis. This study was carried out with the approval of the Ethics Committee (approval number: 2021/05) from the Saki Yenilli Experimental Animal Production and Practice Laboratory.^12^, ^15^, ^16^

### 2.2 Plasma collection

Animals were rendered unconscious by short‐term treatment with ether before blood samples were taken. Pooled rat plasma was collected by intracardial bleed at the time of euthanasia. Plasma was prepared from blood collected with EDTA, followed by centrifugation at 1000g. The plasma was denaturated by heating it for 2–3 min at 95°C, followed by a short spin at 1000 g. All plasma aliquots were stored at −80°C until use. Before it was given, EDTA was taken out of the plasma using 3.5‐kDa D‐tube dialyzers (EMD Millipore) in PBS.^12^

### 2.3 Analysis of samples by attenuated total reflectance Fourier transform infrared (ATR‐FTIR) spectroscopy

Liver samples of all animals (2 × 24 = 48 in total) were compressed on the Zn/Se crystal of the ATR unit (PerkinElmer) without any pretreatment and examined with an ATR‐FTIR spectrometer (PerkinElmer) at a resolution of 4 cm^−1^ and a scan number of 32. The spectra were obtained with the Spectrum One (PerkinElmer) software in the wavelength range of 4000–650 cm^−1^.^17^

### 2.4 Prediction studies with machine learning approach based on big spectral data

Linear Discriminant Analysis (LDA), a machine learning approach, was applied to differentiate the experimental groups. Spectral data were used in pattern recognition analysis. Each sample spectrum was pre‐processed on The Unscrambler® X 10.3 (CAMO Software AS, Norway) software with a baseline offset transformation in the 4000–650 cm^−1^ region to make the analyzes as independent as possible from the FTIR spectrometers. Spectra processed this way were first subjected to Principal Component Analysis (PCA), an unsupervised pattern processing technique.^18^ Spectra were passed from standard deviation normalization (mean centering normalization) and validated using the leverage‐correction method. Subsequently, the spectra were examined in lipid (3000–2700 cm^−1^), protein (1700–1500 cm^−1^), nucleic acid and polysaccharide (1200–650 cm^−1^) and full (4000–650 cm^−1^) regions by Singular Value Decomposition (SVD) algorithm.

LDA is a supervised classifier in which n‐dimensional feature samples are linearly transformed into an m‐dimensional space. PCA only uses sample spectra to determine the transformation, while LDA also uses class information in training samples leading to better classification. PCA data were utilized as LDA model inputs with The Unscrambler® X 10.3 (CAMO Software AS, Norway) multivariate analysis (MVA) software. The category variable column was included in a data matrix, and then all spectra of different sample categories were used to generate a training set. The quadratic method using the projections of the 7 PCA components was used for the prediction. Prior probabilities were calculated from the training set. The results are presented as a discrimination plot, as well as prediction and confusion matrices.^19^

### 2.5 Quantification studies of FTIR spectral bands

Spectral data analysis was performed using OPUS 5.5 (Bruker) software. The average spectra of each sample were baseline corrected using the Rubberband correction method with 64 baseline points before the band quantification analyses. In detailed band analyses, the bands with the highest absorbance values in different spectral regions of the spectra were selected, and the beginning and ending frequencies of the bands were determined with precision. The areas of bands specific to various biomolecules were analysed by taking the integral areas of the determined frequency ranges with the OPUS 5.5 (Bruker) software. In addition, a virtual line was drawn vertically from the band baseline's midpoint to the band's peak, and the length of the line was measured with the help of a virtual ruler. Then, by marking the point where 0.75 times the line length coincides with the line, a horizontal line was drawn along the band from this point, and bandwidth values were obtained.^7^, ^20^

### 2.6 Histopathological analysis

After the dissection of liver specimens, they were fixed in 10% neutral‐buffered formalin at room temperature for 48 h. Paraffin sections with a thickness of 5 μm were prepared using a rotary microtome (Leica Biosystems, Germany). The sections obtained were stained with haematoxylin and eosin stain (haematoxylin: Cas No: 517‐28‐2; Eosin: Cas No.: 17372‐87‐1, Merck, Germany) to visualize the general architecture of the rat liver. The haematoxylin and eosin slides were used to assess the overall tissue characteristics of the rat liver. Histological changes were investigated by randomly evaluating an average of 10–15 areas in each animal section of the groups. The microscopic analysis of the histopathological changes in the study was performed blindly by two researchers.^21^

### 2.7 Haematoxylin and eosin staining

The haematoxylin and eosin staining procedure involved the following sequential steps. First, all slides within each group were deparaffinized by xylene. Subsequently, the slides were dehydrated by passing them through a series of descending ethanol solutions. After dehydration, the slides were immersed in a haematoxylin solution, followed by rinsing under running water. Afterward, the slides were placed in eosin for a duration of 2 min. The next steps included rehydration through a series of ascending ethanol solutions and clearing with xylene.^22^ Finally, all stained slides were mounted with entellan for light microscopy examination.

### 2.8 Masson's trichrome staining

For Masson's trichrome (MT) staining, the instructions provided in kit 04‐010802 from Bio‐Optica (Milan, Italy) were followed. This staining method was used to detect collagen accumulation of fibres in the rat liver. The tissue sections were deparaffinized and rehydrated according to the specific instructions provided in the commercial kit. All the staining procedures were conducted at room temperature.^23^

### 2.9 Quantification of histopathological parameters

To evaluate lymphatic infiltration and microvesicular steatosis (micro lipid droplets) in the rat liver sections, a grayscale binarization approach with the lowest threshold level was employed to distinguish between purple‐stained and non‐stained areas.^24^ The area fractions (%) were quantified using Image J Fiji software (National Institutes of Health, Bethesda, Maryland, USA). For MT staining, a similar grayscale binarization technique with the lowest threshold level was utilized to identify blue‐stained areas, and the MT‐positive area was quantified using Image J Fiji. This quantification method was adapted from the study by Adomshick et al.^25^ Signal intensities from five images within each section were visually presented for all animals in the same group. Analysis of the microphotographs was performed using a light microscopy system comprising a Nikon Eclipse Ni microscope (Tokyo, Japan) with a camera attachment (Nikon DS‐Fi2, Japan), and imaging software (NIS Elements F 4.00.00, Nikon Soft Imaging Solution, Japan) at a magnification of 400×.

### 2.10 Immunohistochemical analysis

Immunohistochemical (IHC) was performed using the previously described protocol for paraffin sections.^21^ After deparaffinization, endogenous peroxidase activity was inhibited by incubation with 3% hydrogen peroxide for 10 min and samples were washed with PBS. Heat‐induced antigen retrieval was performed by incubation in citrate buffer and the sections were incubated in a blocking solution for 10 min to prevent non‐specific binding. After the blocking step, all liver sections were incubated overnight with an anti‐Notch1 (sc‐376403, 1:100, Santa Cruz), anti‐Jagged1 (sc‐390177, 1:100, Santa Cruz) and anti‐Delta1 (sc‐8155, 1:100, Santa Cruz) primer antibodies. The next day, biotinylated antibodies (TP‐125‐BN, Thermo Scientific) and streptavidin peroxidase (TS‐125‐HR) were applied to the sections. Then, a 3,3′‐Diaminobenzidine substrate kit (ab64238, Abcam) was performed for 3–5 min. Finally, all sections were counterstained with Mayer's haematoxylin and visualized using Nikon Eclipse Ni microscope with Nikon DS‐Fi2 camera attachment. Images were imported into ImageJ (Fiji) for quantification of percent areas of Notch1, Jagged1 and Delta1 expression analyses. Notch1, Jagged1 and Delta1 intensity (% Area) measurements in the liver sections were performed over 10 random areas of interest (ROI) (three different sections for each rat per group) adopted by.^26^

### 2.11 Liver biochemical markers

Blood samples obtained from rats were subjected to centrifugation at 3500 rpm and a temperature of +4°C. This centrifugation process led to the separation of serum from the blood components. Subsequently, the serum samples were analysed for the levels of aspartate aminotransferase (AST), alanine aminotransferase (ALT), alkaline phosphatase (ALP), lactate dehydrogenase (LDH) and albumin. The analysis was performed using a Hitachi C502 automated biochemical analyser (Roche, Germany) with commercially available kits from Roche Diagnostics. The enzyme activity of ALT, AST, ALP, LDH and the concentration of albumin were expressed in international units per litre (IU/L) and grams per litre (g/L), respectively.

### 2.12 Statistics

Statistical evaluations and graph plots of the results were made using GraphPad Prism 9 (GraphPad, USA) to analyse the histological and biochemical experimental data. The data were analysed using an unpaired *t*‐test and One‐way ANOVA. The statistical analyses were presented as mean ± standard error of the mean (SEM). To compare the alpha diversities and F/B ratio between the IFpls and control Cnt and Npls groups, an unpaired *t*‐test and one‐way ANOVA (one‐sided *p*‐value) were performed. GraphPad Prism 9 (GraphPad Software, USA) software was used for the comparisons. The level of significance was indicated as *p < 0.05, ***p < 0.001 and ****p < 0.0001. Heatmap analysis of metagenomic counts for bacterial families, genera and species in the groups was conducted using GraphPad Prism 9 (GraphPad Software, USA) software.

## 3 RESULTS

### 3.1 IFpls and Npls caused significant changes in liver biomolecules

**FIGURE 1:**
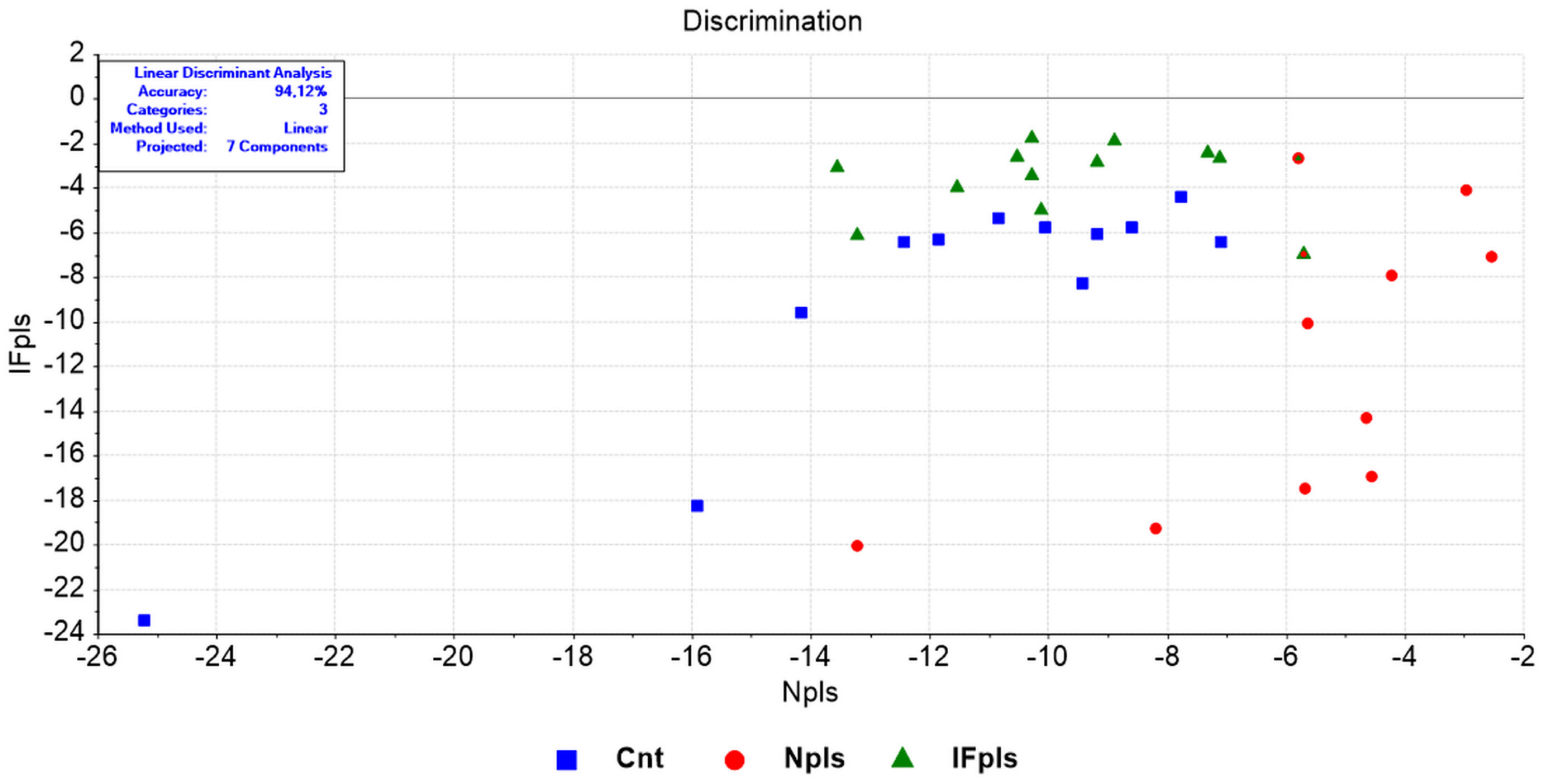
LDA discrimination plot for liver samples in full (4000–650 cm^−1^) spectral region. Cnt (control) group, IFpls (the group receiving plasma from rats undergoing intermittent fasting), and Npls (the group receiving plasma from untreated rats).

**FIGURE 2:**
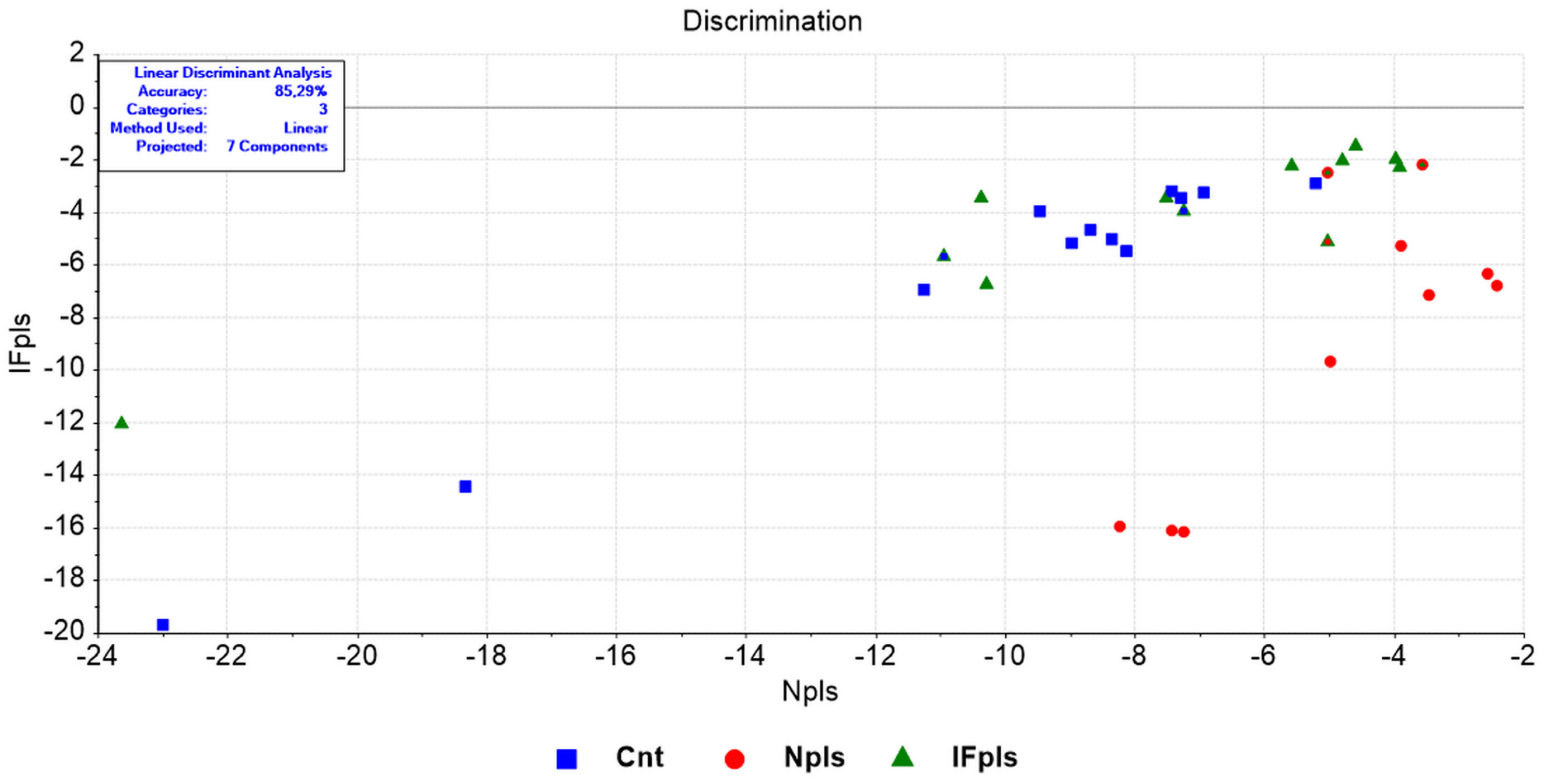
LDA discrimination plot for liver samples in lipid (3000–2700 cm^−1^) spectral region. Cnt (control) group, IFpls (the group receiving plasma from rats undergoing intermittent fasting), and Npls (the group receiving plasma from untreated rats).

LDA application to the dataset achieved impressive accuracy: 94.12% for overall biomolecules (Figure 1; Tables S1 and S2) and 85.12%, 91.18%, and 79.41% for lipids, proteins and nucleic acids, respectively (Figure 2; Figures S1 and S2; Tables S3–S8). Discrimination plots clearly demonstrated unique molecular profiles for each group: the control group (Cnt), the group receiving plasma from rats undergoing intermittent fasting (IFpls), and the group receiving plasma from untreated rats (Npls), emphasizing the specific impact of treatments on liver biomolecules.

**FIGURE 3:**
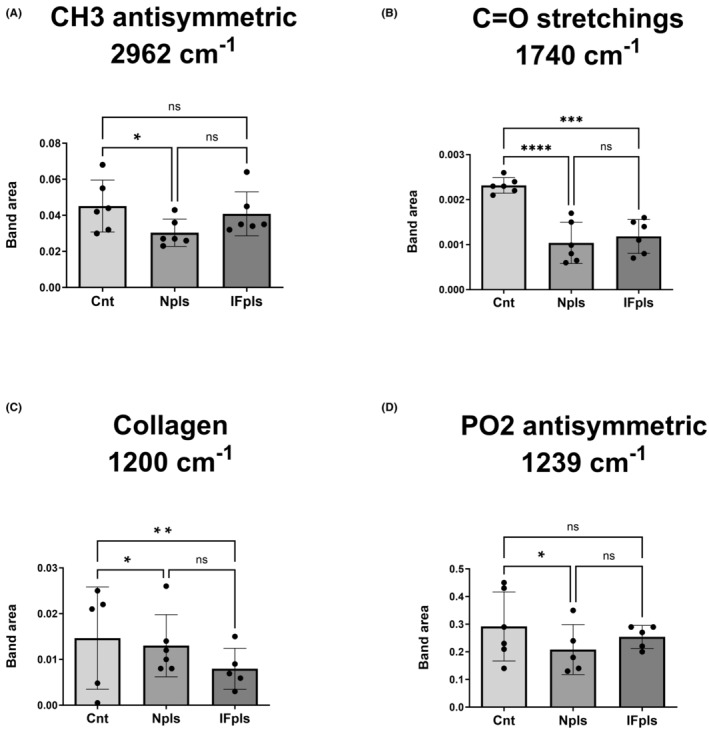
The quantitative changes in lipid, polysaccharide and nucleic acid‐associated spectrochemical parameters. The indices for band area; (A) CH3 antisymmetric stretching (2955 cm^−1^), (B) C = O stretching (1740 cm^−1^), (C) collogen (1200 cm^−1^), and (D) PO2 antisymmetric stretching (1239 cm^−1^). The data were analysed using one‐way ANOVA and unpaired *t*‐test, and significance levels were stated as *p<0.05, **p < 0.01, ***p < 0.001, ****p < 0.0001. Cnt (control) group, IFpls (the group receiving plasma from rats undergoing intermittent fasting), and Npls (the group receiving plasma from untreated rats).

We have observed noticeable alterations in various spectrochemical bands when comparing average spectra (spanning the full infrared region from 4000 to 650 cm^−1^) of liver samples between the control and treatment groups.^27^ In the evaluation of band area, no substantial difference was observed at the CH3 antisymmetric stretching (2955 cm^−1^) band, typically associated with lipids and proteins content, following IFpls treatment (Figure 3A). Nevertheless, this band index showed a considerable decrease post‐Npls treatment. The band at 1740 cm^−1^, corresponding to C = O stretching and used as an indicator of cholesterol ester, displayed a significant reduction in both IFpls and Npls treatment groups when compared to the control group (Figure 3B). Notably, the decrease was more substantial following Npls treatment. The band associated with collagen (1200 cm^−1^) showed a marked reduction in both treatment groups as compared to the control, with a larger decrease observed post‐IFpls treatment (Figure 3C). The decrease in the value of the PO2 antisymmetric stretching (1239 cm^−1^) band, used for assessing nucleic acids, was significant only following Npls transfusion (Figure 3D).

**FIGURE 4:**
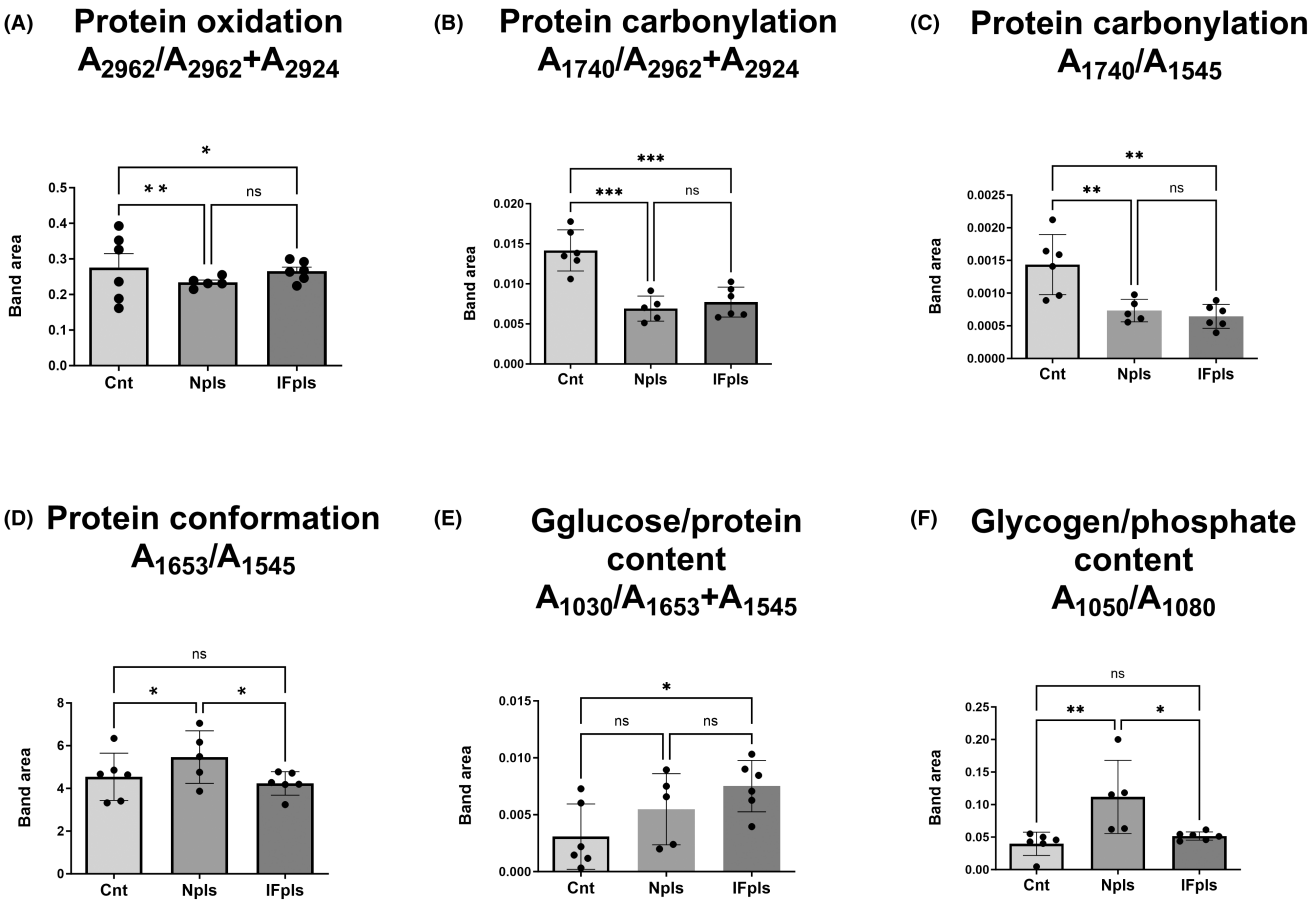
The quantitative changes in lipid, polysaccharide, and nucleic acid‐associated spectrochemical parameters. The indices for band area ratio; (A) protein oxidation (A_2962_/A_2962+2924_), (B) protein carbonylation (A_1740_/A_2962+2924_), (C) protein carbonylation (A_1740_/A_1545_), (D) protein conformation (A_1653_/A_1545_), (E) glucose/protein content (A_1030_/A_1653+1545_), and (F) glycogen/phosphate content (A_1050_/A_1080_). The data were analysed using one‐way ANOVA and unpaired *t*‐test, and significance levels were stated as *p < 0.05, **p < 0.01, ***p < 0.001. A (Absorbance), Cnt (control) group, IFpls (the group receiving plasma from rats undergoing intermittent fasting) and Npls (the group receiving plasma from untreated rats).

Based on the data analysed, it is evident that the effects of protein oxidation, protein carbonylation, protein conformation, glucose/protein content and glycogen/phosphate content differ between the two treatment groups and the control group. Considering the band area ratios of protein oxidation (A_2962_/A_2962+A2924_), a marked decrease was observed in both treatment groups when compared to the control, with the Npls treatment demonstrating a more pronounced decrease (Figure 4A). This suggests that both treatment methods might have similar effects on reducing protein oxidation, with Npls seemingly having a superior impact. Protein carbonylation, assessed via the band area ratios (A_1740_/A_2962+2924_) and (A_1740_/A_1545_), showed significant reductions in both treatment groups compared to the control (Figure 4B,C). However, no substantial difference was found between the IFpls and Npls treatments, implying that both treatments were equally effective in mitigating protein carbonylation. In terms of protein conformation (A_1653_/A_1545_), a notable increase was seen following the Npls treatment compared to the control (Figure 4D). On the other hand, IFpls transfusion did not result in a significant difference. For glucose/protein content (A_1030_/A_1653+1545_), there was a significant increase only with the IFpls transfusion compared to the control (Figure 4E). The increase with the Npls treatment did not result in a significant difference, suggesting that IFpls transfusion may be more efficient in enhancing glucose/protein content. Lastly, glycogen/phosphate content (A_1050_/A_1080_) showed a significant increase after Npls treatment compared to both the control and IFpls (Figure 4F). Interestingly, the increase observed after IFpls was less significant than the control, indicating that Npls treatment could potentially be more effective in promoting glycogen/phosphate content.

Analysis of the spectral bandwidth has also yielded significant findings. The CH2 antisymmetric band at 2922 cm^−1^ displayed a significant increase after the IFpls treatment, when compared to both the control and Npls treatments (Figure S3A). Importantly, this increase was more pronounced when compared to the Npls treatment, suggesting a differential impact of the IFpls treatment on CH2 antisymmetric band intensity. On the other hand, the amide I band at 1653 cm^−1^ exhibited a significant decrease exclusively with the Npls treatment, when compared to the control group (Figure S3B). This indicates a unique influence of the Npls treatment on the amide I band, specifically causing its decrease.

### 3.2 Effects of Npls and IFpls treatments on aged liver histology

**FIGURE 5:**
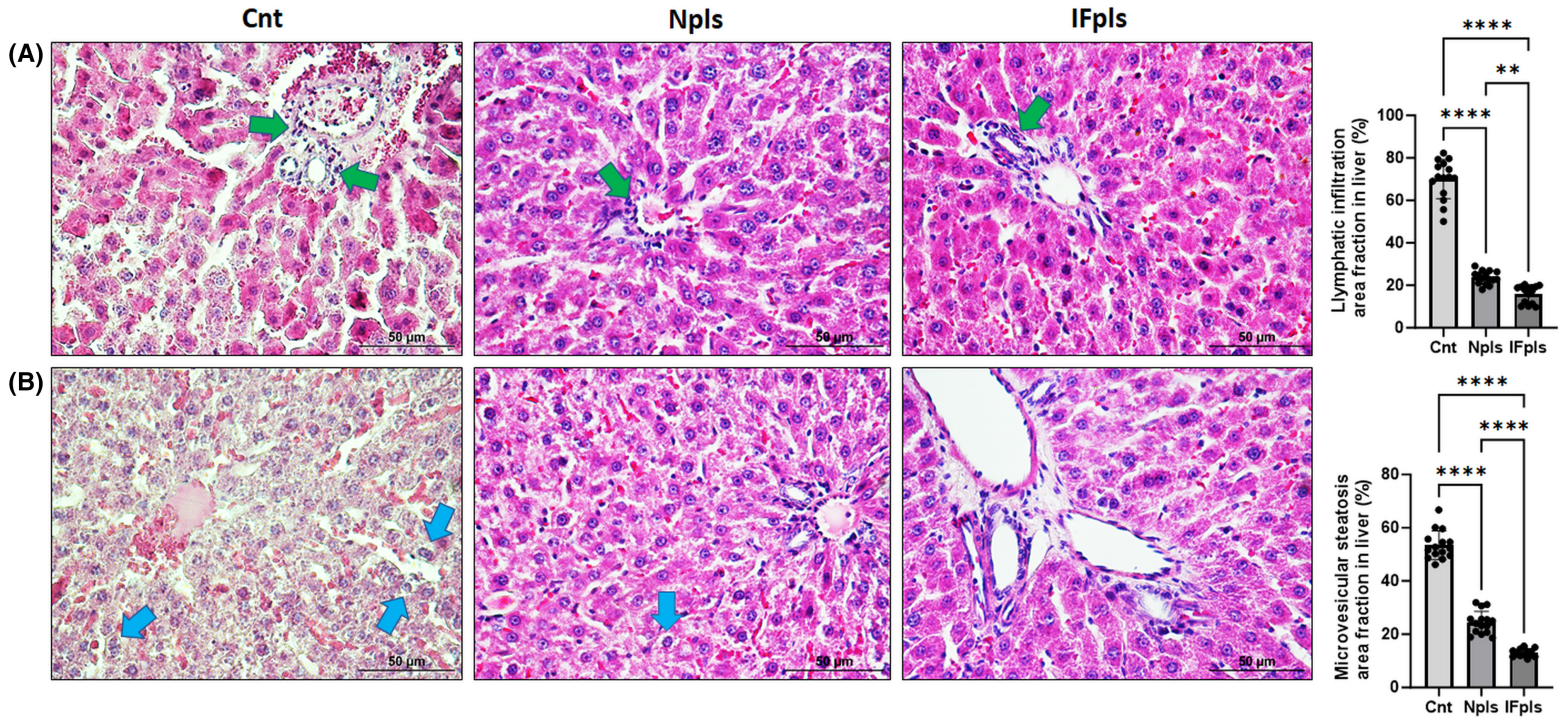
Aged rat livers (Cnt) show increased steatosis and lymphatic infiltration, but Npls and IFpls groups improved these histopathological alterations. (A) Representative images of haematoxylin and eosin staining and quantification of lymphatic infiltration area fraction (%) in all groups. (B) Representative images of haematoxylin and eosin staining and quantification of hepatic microvesicular steatosis area fraction (%). Green arrows show lymphatic infiltrates. Blue arrows show microvesicular steatosis. Values are expressed as mean ± SEM; *n* = 7 rats in each group. *p* ≤ 0.01**, and *p* ≤ 0.0001**** (nonparametric Mann–Whitney *U*‐test). Scale bar = 50 μm. Cnt (control) group, IFpls (the group receiving plasma from rats undergoing intermittent fasting) and Npls (the group receiving plasma from untreated rats).

The study investigated the histopathological effects of Npls and IFpls on aged liver structural alterations. As shown in Figure 5, control group exhibited fibrosis, tissue damage, irregular hepatocyte arrangement, increased sinusoidal areas along with intense lymphatic infiltration. In contrast, in both treatment groups displayed normal liver architecture. Npls and IFpls administration significantly improved hepatic fibrosis, cellular degeneration and lymphatic infiltration in treatment groups compared to the control group. IFpls significantly appeared to reduce age‐related inflammation in the aged liver (see Figure 5A) indicating the potential of these treatments in alleviating age‐related hepatic damage.

### 3.3 Npls and IFpls suppress fat accumulation in hepatocytes in aged rats

This study investigated the effects of Npls and IFpls treatments on microvesicular steatosis, a form of hepatic fat deposition, in the aged‐related livers. Histological examination revealed hepatocyte nuclei condensation, nuclear membrane changes and fragmentation in control group compared to the treatment groups. However, Npls and IFpls groups showed a significant reduction in the density of fat droplets compared to the control group (see Figure 5B). The administration of Npls and IFpls treatments to aged rats led to a substantial decrease in hepatic microvesicular steatosis, suggesting their potential in mitigating age‐related liver changes.

### 3.4 Npls and IFpls treatments reduces hepatic collagen accumulation in age‐related fibrosis

**FIGURE 6:**
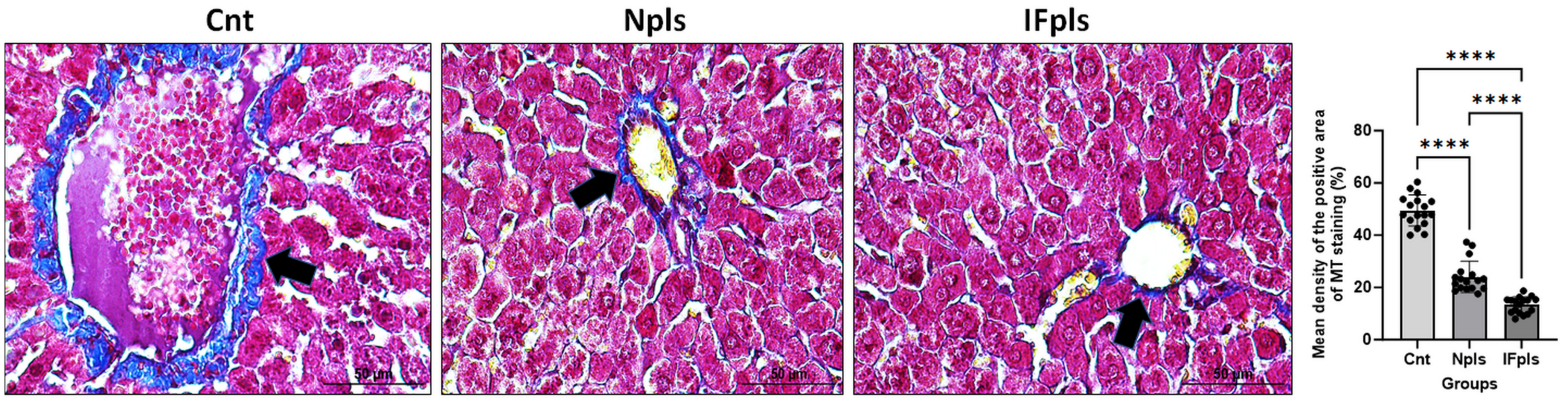
Representative images of MT staining showing collagen deposition in Cnt, Npls and IFpls groups liver tissue with quantification of collagen density area fraction (%) in all groups. Black arrows show collagen depositions. Values are expressed as mean ± SEM; *n* = 7 rats in each group. *p* ≤ 0.0001**** (nonparametric Mann–Whitney U‐test) versus control. Scale bar = 50 μm. Cnt (control) group, IFpls (the group receiving plasma from rats undergoing intermittent fasting) and Npls (the group receiving plasma from untreated rats).

To determine whether Npls and IFpls treatments alleviate aging‐related liver fibrosis, collagen deposition was measured in the liver sections of all groups by using MT staining. The results showed that blue positive areas were significantly decreased in Npls and IFpls groups compared to the control group. However, when compared to the control group, the levels of collagen deposition density were more significantly reduced in the IFpls groups, suggesting their potential in alleviating age‐related liver fibrosis (see Figure 6).

### 3.5 Npls and IFpls downregulates Notch signalling contributing to inflammation and liver fibrosis in aged rats

**FIGURE 7:**
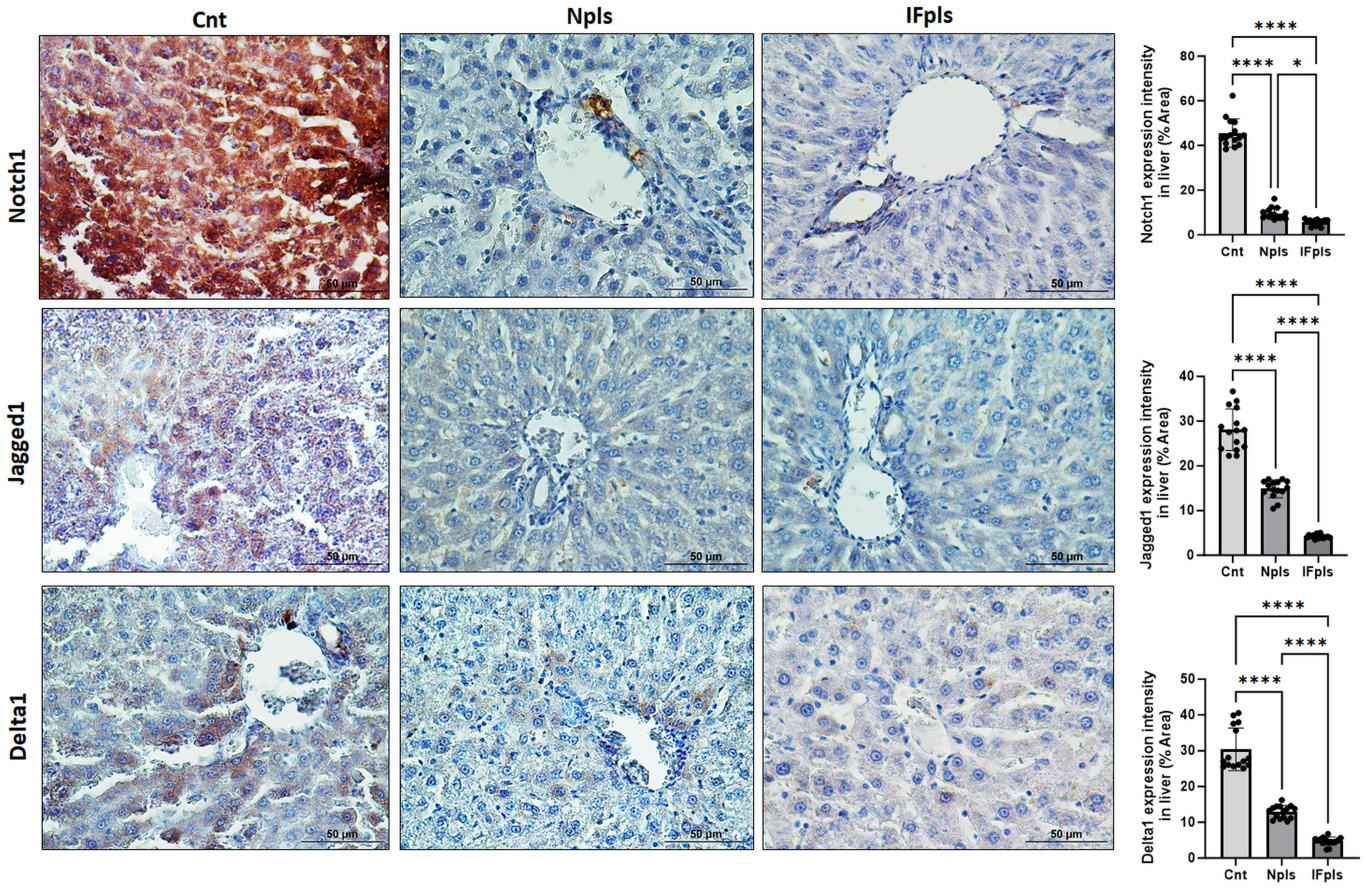
Notch1, Jagged 1 and Delta1 staining intensity in rat liver. IHC staining images of Notch1, Jagged 1 and Delta1 expressions in all groups. Graphs of Notch1, Jagged 1 and Delta1 staining intensity in the liver as measured in ImageJ (FIJI). Cnt (control) group, IFpls (the group receiving plasma from rats undergoing intermittent fasting), and Npls (the group receiving plasma from untreated rats); (*p < 0.05 and ****p ≤ 0.0001) (IHC staining, Scale bar: 50 μm).

Notch signalling has a great impact on the occurrence and development of hepatic fibrosis and can interact with aged‐related inflammation process. The Notch signalling pathway is activated when the extracellular domain of the Notch receptor is bound by a ligand, for example, Jagged or Delta, on neighbouring cells. So, IHC staining was performed to evaluate the effect of Npls and IFpls treatment on Notch1, Jagged 1 and Delta1 expressions in aged rats for evaluating liver alterations associated with aging inflammation. In the results of the study, Notch1, Jagged 1 and Delta1 immunoreactivity was markedly increased in the cytoplasmic areas of hepotocytes for the control group compared to Npls and IFpls groups (*p* < 0.0001, Figure 7). However, a significant decrease in Notch1, Jagged 1 and Delta1 staining intensity was detected in Npls and IFpls gorup compared to the control group (*p* < 0.0001). Interestingly, the decrease in Notch1, Jagged 1 and Delta1 staining intensity in IFpls was statistically significant compared to Npls (see Figure 7).

### 3.6 Evaluation of the serum liver enzymes markers in aged rats treated with Npls and IFpls

The results showed that serum ALP levels increased in the IFpls group compared to control and Npls group, while ALT and AST levels showed no significant differences between control group and IFpls group (see Figure S4A–S4C). Metabolic liver adaptations related with aging process are not well understood, but biochemical changes in LDH and albumin levels have been linked to liver diseases. There were no significant differences between control group and IFpls group for LDH levels. LDH, an essential enzyme found primarily in muscle, liver and kidney cells, showed increased serum levels in the Npls group compared to the control group (Figure S4D). A decrease in serum albumin concentration with aging has been previously reported, in this study that IFpls group had significantly increased serum albumin levels compared to control and Npls groups (Figure S4E).

## 4 DISCUSSION

This research sheds light on the influence of plasma derived from intermittently fasting 12‐month‐old rats (IFpls) and untreated rats (Npls) on age‐related liver diseases in 24‐month‐old rats. Specifically, this study is one of the first to quantitatively assess the influence of IFpls and Npls on liver biomolecules and histology, highlighting distinct protective effects against hepatic instability, microvesicular steatosis and liver fibrosis. Furthermore, this research underscores the potential of leveraging dietary regimens to modulate the biochemical landscape of plasma, thereby influencing systemic aging processes and offering a non‐invasive approach to mitigate the effects of aging on liver health.

The beneficial effects of IFpls were particularly notable, displaying a decrease in liver instability, microvesicular steatosis and fibrosis. These findings indicate that the metabolic and biochemical adjustments induced by intermittent fasting, such as increased autophagy and improved metabolic flexibility, may extend beyond general health improvements to provide specific benefits in preventing liver aging. This aligns with the hypothesis that intermittent fasting can regulate systemic inflammatory responses and oxidative stress, conditions that are strongly associated with the development of liver diseases.^1^, ^5^ Supporting this, a recent study found similar effects in rats that underwent intermittent fasting for 30 days. The study analysed liver tissues from 24‐month‐old Sprague–Dawley rats and demonstrated substantial reductions in hepatocyte degeneration, lymphocytic infiltration, steatosis and fibrosis, which aligns with our findings.^28^ Additionally, our research team conducted another investigation that involved infusing plasma derived from 5‐week‐old young rats into 24‐month‐old aged rats. The results indicated that young plasma infusion significantly improved hepatic fibrosis and cellular degeneration, and markedly reduced hepatic microvesicular steatosis in the aged rats.^24^ These observations suggest that intermittent fasting may rejuvenate the plasma factors to resemble those found in much younger rats, potentially reversing some aging markers in plasma composition. However, the present investigation did not undertake an examination of the plasma constituents in IFpls and Npls. This limitation highlights the need for further research to accurately interpret these outcomes and fully understand the implications of fasting‐induced changes in plasma composition for liver health and overall aging.

In contrast, the administration of Npls demonstrated a significant reduction in protein oxidation and changes in biomolecular content, highlighting its potential to facilitate cellular rejuvenation. This observation aligns with prior research suggesting that young plasma contains beneficial factors for tissue repair and anti‐aging at the cellular level.^9^, ^10^ Additionally, the decrease in protein carbonylation—a biomarker of oxidative stress and aging—across both Npls and IFpls treatments suggests a possible common pathway through which these plasma treatments may exert their protective effects, likely associated with their impact on systemic redox states.^29^

However, it is important to note that the lesser reduction in protein oxidation observed in the IFpls group compared to the Npls group does not necessarily indicate superiority of IFpls. Rather, it suggests potential differences in the mechanisms through which these plasma types affect aging‐related pathophysiology, where protein oxidation plays a critical role in mediating physiological events and mitigating tissue damage.^29^ The marked improvement in protein conformation following Npls treatment also warrants further investigation to fully understand its implications. Such an inquiry would require advanced proteomic techniques or comparable methodologies to elucidate the underlying mechanisms and potential impacts on cellular function and physiology, thereby offering a deeper insight into how plasma from young and intermittently fasting rats can differentially influence aging processes at the molecular level. In addition, the differential impact on lipid and nucleic acid profiles by IFpls and Npls underscores the complexity of how different forms of plasma influence liver biochemistry. These findings require further investigation into the specific components of IF‐induced plasma that confer a distinct advantage over untreated plasma, potentially involving factors related to enhanced lipolysis and improved protein homeostasis observed during fasting periods.^30^

The relationship between aging and the development of hepatic inflammation, fibrosis and steatosis has been extensively documented in both human and rodent studies.^31^, ^32^, ^33^ These conditions can lead to structural deterioration in the aging liver and elevate the overall metabolic risk. Npls and IFpls have demonstrated potential in mitigating age‐related liver damage, likely due to their antioxidant, anti‐inflammatory and immunomodulatory properties, which may enhance the regenerative capacity of hepatocytes. This regenerative capacity is crucially supported by the activation of morphogenic signalling pathways like Notch and Hedgehog, which facilitate phenotypic changes in hepatic stellate cells (HSCs) and drive transitions between epithelial and mesenchymal states.^34^, ^35^, ^36^

Notably, the activation of the Notch signalling pathway is generally upregulated to regenerate new hepatocytes and HSCs in aged rats, which can combat the effects of physiological changes such as inflammation, steatosis and fibrosis. However, overactivation of this pathway might also promote fibrosis by increasing inflammation, which can exacerbate liver damage.^37^, ^38^ Our findings suggest that while increased Notch signalling during aging can contribute to lipid accumulation and fibrosis, the application of Npls and IFpls can significantly modulate this pathway, thereby aiding in the preservation and improvement of liver architecture and function.

Furthermore, the evaluation of fibrosis, a prevalent age‐related alteration in various organs, particularly the liver, was conducted using MT staining. This technique highlighted an increase in collagen density, a key indicator of fibrosis, in the control group's liver tissues.^39^ In contrast, both IFpls and Npls treatments were effective in reducing collagen density, suggesting a protective role against the progression of liver fibrosis. Notably, the plasma derived from rats undergoing intermittent fasting exhibited a more pronounced effect in suppressing liver fibrosis, which underscores the potential of IF‐induced modifications in plasma to confer significant protective benefits against age‐related hepatic changes.

## 5 CONCLUSSION

The research examined the significant influence of plasma from 12‐month‐old intermittently fasting rats (IFpls) and untreated rats (Npls) on the molecular and histological changes in the liver of aged Sprague–Dawley rats. The IFpls demonstrated a protective effect by reducing hepatic instability, microvesicular steatosis and fibrosis and by lowering the expression of inflammation‐associated proteins Notch1, Jagged1 and Delta1. Both IFpls and Npls were effective in diminishing protein oxidation and carbonylation, with Npls particularly enhancing protein conformation and increasing glycogen/phosphate content, while IFpls uniquely boosted glucose/protein content. These results highlight the distinct biochemical effects of both treatments, providing crucial insights for potential therapeutic strategies against liver disease and aging.

However, the study's reliance on an animal model limits the direct applicability of findings to human physiology, and the focus on specific biomarkers may overlook broader systemic effects. The small sample size and short duration of the treatments further constrain the generalizability of the results, indicating a need for extended research with larger groups to fully ascertain the long‐term impacts of these interventions.

Future research should focus on translating these findings into clinical trials to evaluate the efficacy and safety of plasma treatments in human liver diseases linked to aging. Identifying the specific active components in IFpls that confer protective benefits will be vital, as will exploring the broader systemic implications of these plasma treatments, including their effects on immune function and metabolic processes.

## AUTHOR CONTRIBUTIONS

**Hüseyin Allahverdi:** Conceptualization (equal); data curation (equal); formal analysis (equal); funding acquisition (equal); investigation (equal); methodology (equal); resources (equal); software (equal); supervision (equal); validation (equal); visualization (equal); writing – original draft (equal); writing – review and editing (equal).

## FUNDING INFORMATION

No financial support was received for this study.

## CONFLICT OF INTEREST STATEMENT

The author has declared that no competing interests exist.

## Supporting information


Figure S1.

Table S1.

## Data Availability

All data generated during and/or analyzed during the current study are available from the corresponding author upon reasonable request.
