# Supplementary material for: Exploring the therapeutic potential of plasma from intermittent fasting and untreated rats on aging‐induced liver damage
Source: J Cell Mol Med. 2024 Jun 25;28(12):e18456. doi: 10.1111/jcmm.18456 (PMC11199341; doi:10.1111/jcmm.18456)
Supplement: Supplementary file 1 — Figure S1. [file JCMM-28-e18456-s001.docx]

**SUPPLEMENTARY FIGURES**

**
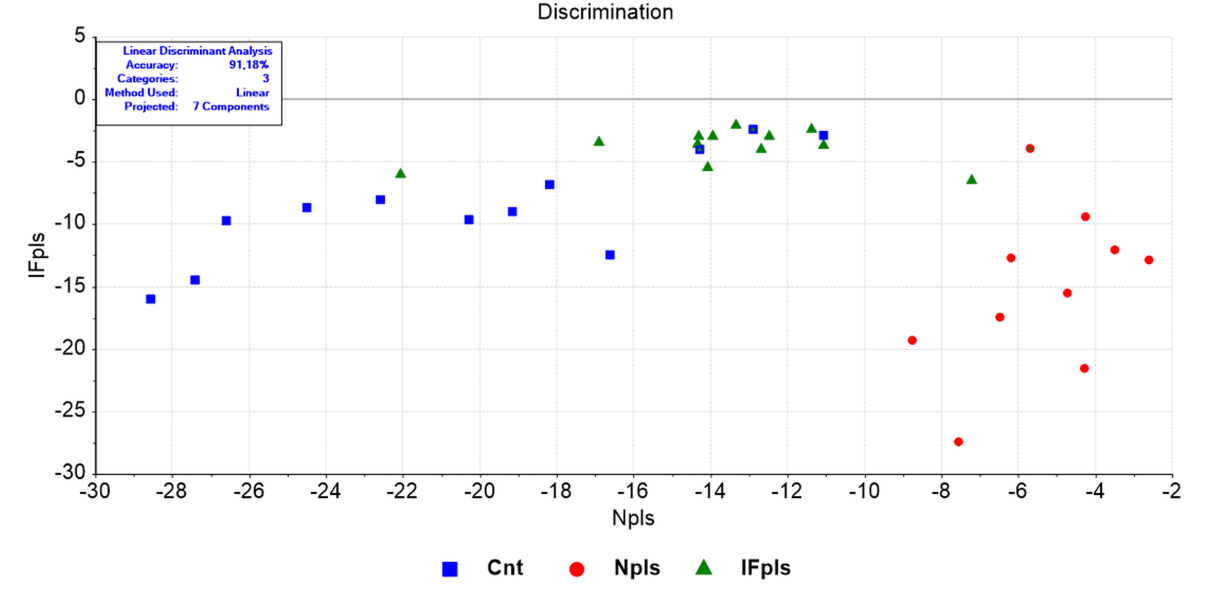
**

**Fig. S1** LDA discrimination plot for liver samples in protein (1700-1500 cm^-1^) spectral region. Cnt (control), IFpls (the group receiving plasma from rats undergoing intermittent fasting), and Npls (the group receiving plasma from untreated rats).


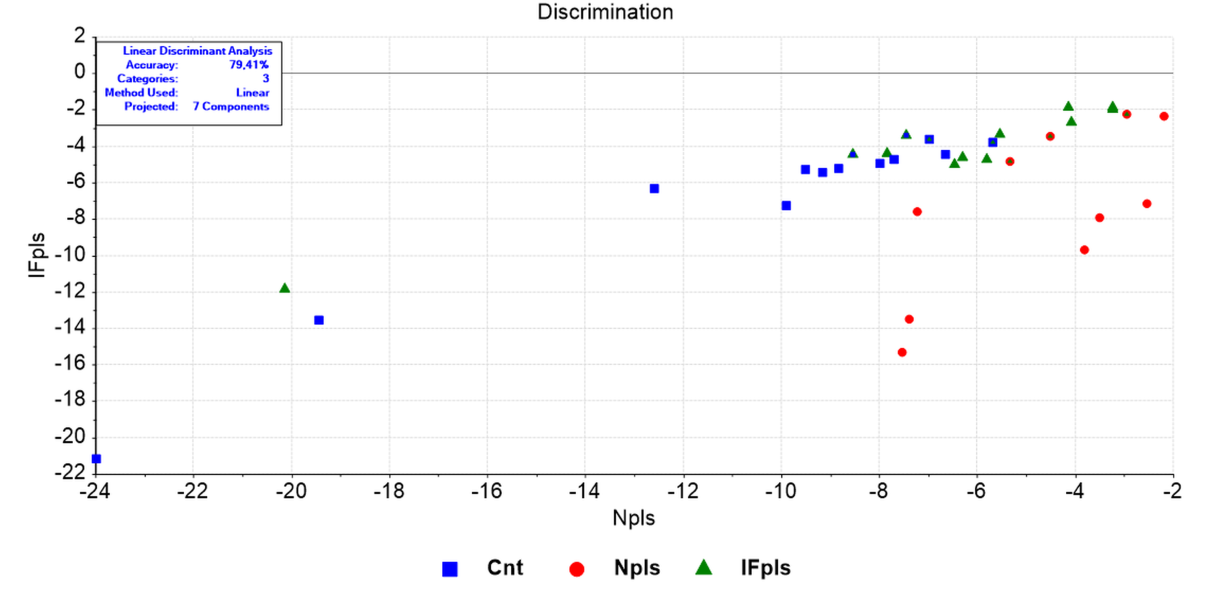


**Fig. S2** LDA discrimination plot for liver samples in spectral region in nucleic acids and polysaccharides (1200-650 cm^-1^). Cnt (control), IFpls (the group receiving plasma from rats undergoing intermittent fasting), and Npls (the group receiving plasma from untreated rats).


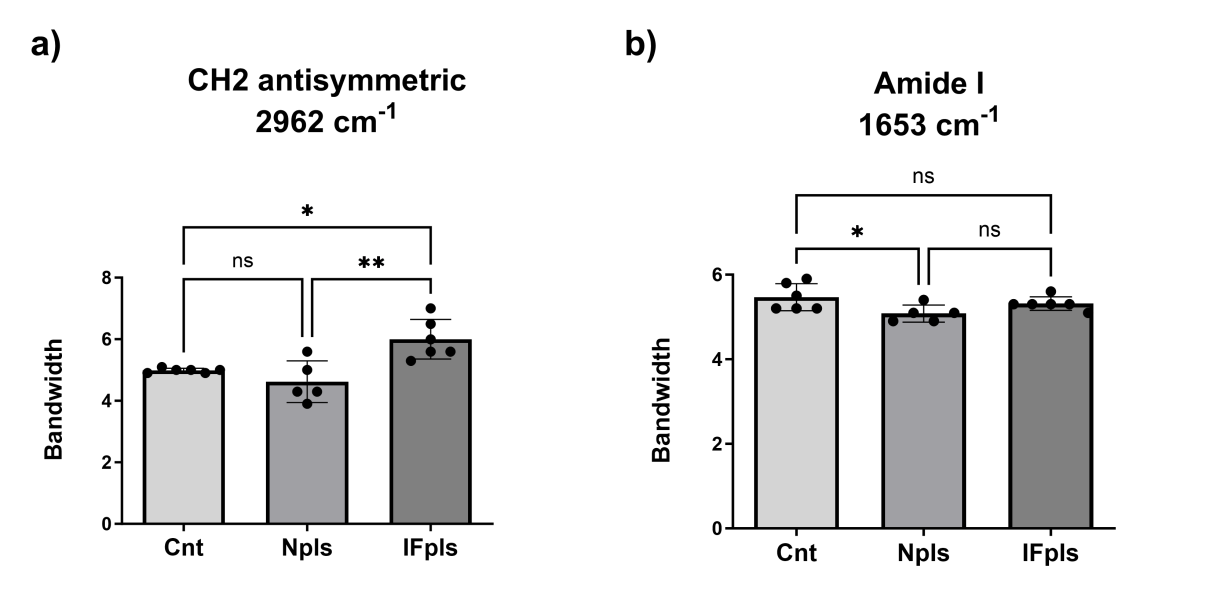


**Fig. S3** The quantitative changes in bandwidth spectrochemical parameters. The indices for **a)** 2962 cm^-1^ (CH_2_ antisymmetric stretching: lipids and proteins), and **b)** 1653 cm^-1^ (Amide I: *α-*helical structure of proteins). Cnt (control), IFpls (the group receiving plasma from rats undergoing intermittent fasting), and Npls (the group receiving plasma from untreated rats).


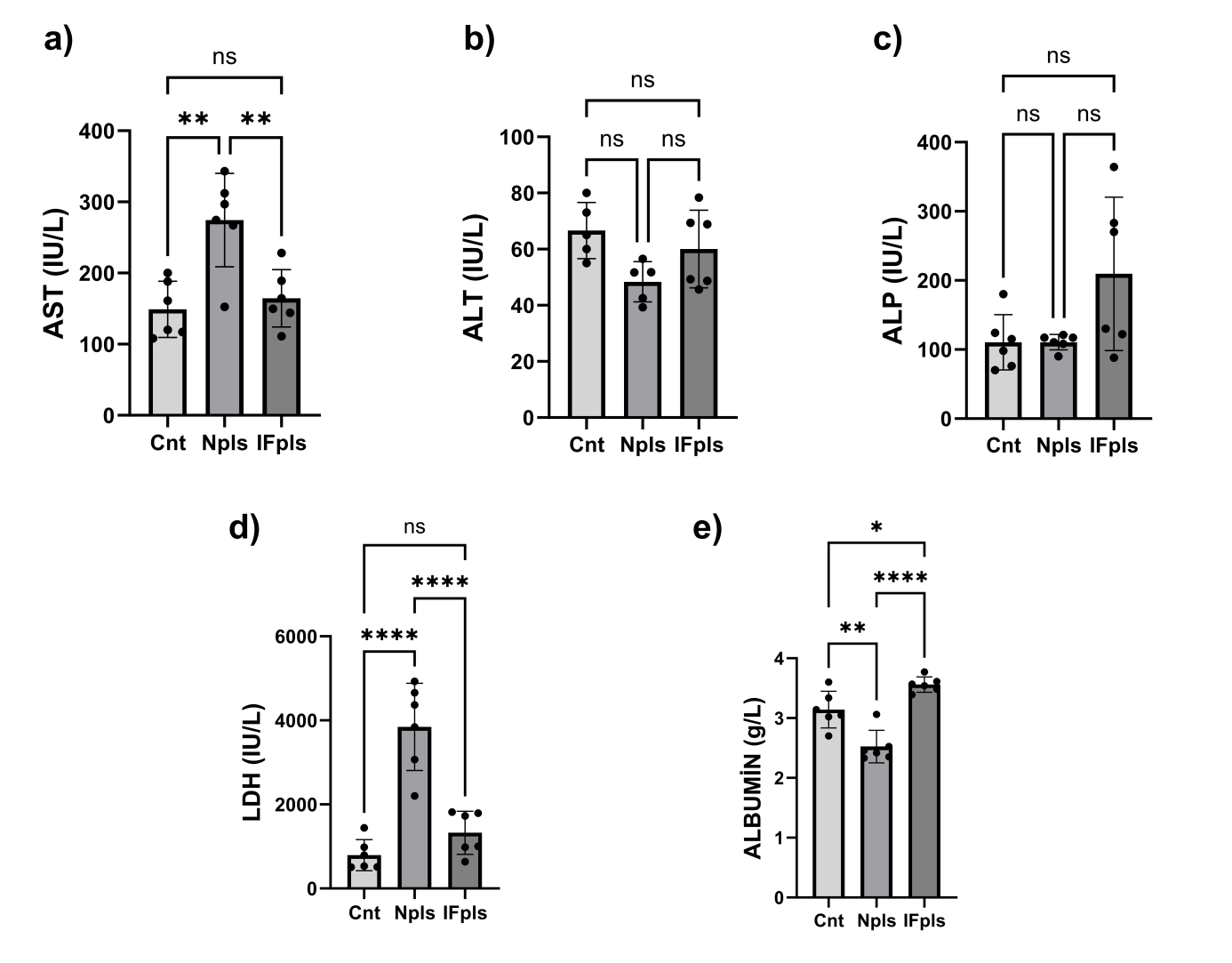


**Fig. S4** Effects of plasma from rats undergoing intermittent fasting and the group receiving plasma from untreated rats on serum **a**) AST, **b**) ALT, **c**) ALP, **d**) LDH, and **e**) Albumin levels in IFpls, Npls, and Cnt groups. Values are expressed in Mean ± SE. n = 6, *p < 0.05 compared with Cnt (control), IFpls (the group receiving plasma from rats undergoing intermittent fasting), and Npls (the group receiving plasma from untreated rats).
