# Supplementary material for: Exploring the therapeutic potential of plasma from intermittent fasting and untreated rats on aging‐induced liver damage
Source: J Cell Mol Med. 2024 Jun 25;28(12):e18456. doi: 10.1111/jcmm.18456 (PMC11199341; doi:10.1111/jcmm.18456)
Supplement: Supplementary file 2 — Table S1. [file JCMM-28-e18456-s002.docx]

**SUPPLEMENTARY TABLES**

**Table S1** LDA confusion matrix for liver samples in the full (4000-650 cm^-1^) spectral region. Cnt (control), IFpls (the group receiving plasma from rats undergoing intermittent fasting), and Npls (the group receiving plasma from untreated rats).

| **Confusion matrix** | **Actual** | **Cnt** | **Npls** | **IFpls** |
| --- | --- | --- | --- | --- |
| Predicted |  | 1 | 2 | 3 |
| **Cnt** | 1 | **12** | 0 | 0 |
| **Npls** | 2 | 0 | **10** | 0 |
| **IFpls** | 3 | 0 | 0 | **12** |

**Table S2** LDA confusion matrix for liver samples in the full (4000-650 cm^-1^) spectral region. Cnt (control), IFpls (the group receiving plasma from rats undergoing intermittent fasting), and Npls (the group receiving plasma from untreated rats).

|  | **Cnt** | **Npls** | **IFpls** | **Predicted** |
| --- | --- | --- | --- | --- |
|  | 1 | 2 | 3 | 4 |
| 1 | -9,22 | -15,63 | -13,82 | Cnt |
| 2 | -2,11 | -18,02 | -21,96 | Cnt |
| 3 | -4,52 | -14,60 | -15,47 | Cnt |
| 4 | -3,59 | -15,69 | -17,30 | Cnt |
| 5 | -3,37 | -20,57 | -16,36 | Cnt |
| 6 | -5,53 | -28,94 | -24,78 | Cnt |
| 7 | -2,82 | -15,84 | -21,94 | Cnt |
| 8 | -2,70 | -19,25 | -22,61 | Cnt |
| 9 | -2,55 | -18,57 | -21,90 | Cnt |
| 10 | -2,35 | -20,23 | -21,11 | Cnt |
| 11 | -10,37 | -27,37 | -29,28 | Cnt |
| 12 | -2,86 | -24,87 | -23,09 | Cnt |
| 13 | -15,97 | -2,32 | -28,12 | Npls |
| 14 | -21,33 | -2,74 | -33,39 | Npls |
| 15 | -27,54 | -4,76 | -41,82 | Npls |
| 16 | -36,08 | -7,47 | -32,35 | Npls |
| 17 | -16,74 | -4,08 | -18,02 | Npls |
| 18 | -13,74 | -4,78 | -21,54 | Npls |
| 19 | -14,99 | -2,75 | -26,80 | Npls |
| 20 | -16,18 | -3,45 | -23,61 | Npls |
| 21 | -16,18 | -3,79 | -28,41 | Npls |
| 22 | -18,72 | -5,00 | -30,22 | Npls |
| 23 | -16,69 | -9,72 | -9,03 | IFpls |
| 24 | -14,77 | -16,92 | -2,86 | IFpls |
| 25 | -17,66 | -22,43 | -3,09 | IFpls |
| 26 | -13,59 | -26,72 | -6,08 | IFpls |
| 27 | -23,18 | -30,68 | -2,72 | IFpls |
| 28 | -22,63 | -34,50 | -2,29 | IFpls |
| 29 | -34,42 | -38,89 | -5,45 | IFpls |
| 30 | -25,69 | -34,02 | -2,84 | IFpls |
| 31 | -14,56 | -26,68 | -3,22 | IFpls |
| 32 | -24,20 | -38,37 | -7,32 | IFpls |
| 33 | -25,31 | -35,30 | -4,37 | IFpls |
| 34 | -17,68 | -30,29 | -3,49 | IFpls |

**Table S3** LDA confusion matrix for liver samples in lipid (3000-2700 cm^-1^) spectral region. Cnt (control), IFpls (the group receiving plasma from rats undergoing intermittent fasting), and Npls (the group receiving plasma from untreated rats).

| **Confusion matrix** | **Actual** | **Cnt** | **Npls** | **IFpls** |
| --- | --- | --- | --- | --- |
| Predicted |  | 1 | 2 | 3 |
| **Cnt** | 1 | **11** | 0 | 0 |
| **Npls** | 2 | 0 | **10** | 0 |
| **IFpls** | 3 | 1 | 0 | **12** |

**Table S4** LDA confusion matrix for liver samples in lipid (3000-2700 cm^-1^) spectral region. Cnt (control), IFpls (the group receiving plasma from rats undergoing intermittent fasting), and Npls (the group receiving plasma from untreated rats).

|  | **Cnt** | **Npls** | **IFpls** | **Predicted** |
| --- | --- | --- | --- | --- |
|  | 1 | 2 | 3 | 4 |
| 1 | -7,04 | -19,31 | -6,69 | IFpls |
| 2 | -2,35 | -34,37 | -8,98 | Cnt |
| 3 | -4,32 | -25,93 | -4,95 | Cnt |
| 4 | -4,08 | -25,96 | -4,82 | Cnt |
| 5 | -2,90 | -28,63 | -5,88 | Cnt |
| 6 | -3,52 | -29,46 | -5,26 | Cnt |
| 7 | -2,45 | -35,89 | -10,68 | Cnt |
| 8 | -2,99 | -37,56 | -11,93 | Cnt |
| 9 | -2,71 | -37,99 | -11,06 | Cnt |
| 10 | -2,45 | -32,33 | -9,53 | Cnt |
| 11 | -10,84 | -46,45 | -18,44 | Cnt |
| 12 | -2,40 | -32,64 | -9,31 | Cnt |
| 13 | -32,51 | -3,59 | -16,30 | Npls |
| 14 | -30,42 | -2,36 | -15,21 | Npls |
| 15 | -29,38 | -5,20 | -13,57 | Npls |
| 16 | -48,25 | -7,24 | -24,76 | Npls |
| 17 | -31,13 | -9,39 | -16,60 | Npls |
| 18 | -18,57 | -5,60 | -6,43 | Npls |
| 19 | -46,54 | -6,36 | -27,92 | Npls |
| 20 | -46,39 | -5,26 | -27,32 | Npls |
| 21 | -21,40 | -7,73 | -10,65 | Npls |
| 22 | -34,58 | -4,36 | -19,54 | Npls |
| 23 | -12,32 | -14,63 | -4,74 | IFpls |
| 24 | -9,74 | -13,70 | -2,05 | IFpls |
| 25 | -8,30 | -13,53 | -2,95 | IFpls |
| 26 | -9,35 | -14,50 | -3,42 | IFpls |
| 27 | -9,63 | -12,88 | -2,44 | IFpls |
| 28 | -8,18 | -16,37 | -2,46 | IFpls |
| 29 | -5,77 | -19,89 | -2,95 | IFpls |
| 30 | -6,84 | -15,26 | -2,34 | IFpls |
| 31 | -8,96 | -19,17 | -4,02 | IFpls |
| 32 | -5,51 | -21,82 | -5,44 | IFpls |
| 33 | -7,61 | -14,19 | -4,70 | IFpls |
| 34 | -7,96 | -10,18 | -3,21 | IFpls |

**Table S5** LDA confusion matrix for liver samples in protein (1700-1500 cm^-1^) spectral region. Cnt (control), IFpls (the group receiving plasma from rats undergoing intermittent fasting), and Npls (the group receiving plasma from untreated rats).

| **Confusion matrix** | **Actual** | **Cnt** | **Npls** | **IFpls** |
| --- | --- | --- | --- | --- |
| Predicted |  | 1 | 2 | 3 |
| **Cnt** | 1 | **12** | 0 | 0 |
| **Npls** | 2 | 0 | **10** | 0 |
| **IFpls** | 3 | 0 | 0 | **12** |

**Table S6** LDA confusion matrix for liver samples in protein (1700-1500 cm^-1^) spectral region. Cnt (control), IFpls (the group receiving plasma from rats undergoing intermittent fasting), and Npls (the group receiving plasma from untreated rats).

|  | **Cnt** | **Npls** | **IFpls** | **Predicted** |
| --- | --- | --- | --- | --- |
|  | 1 | 2 | 3 | 4 |
| 1 | -5,88 | -16,68 | -12,81 | Cnt |
| 2 | -3,46 | -15,28 | -9,94 | Cnt |
| 3 | -4,22 | -23,66 | -18,07 | Cnt |
| 4 | -3,87 | -22,17 | -16,98 | Cnt |
| 5 | -3,34 | -29,68 | -17,27 | Cnt |
| 6 | -5,48 | -32,38 | -20,91 | Cnt |
| 7 | -3,05 | -21,43 | -11,69 | Cnt |
| 8 | -2,76 | -16,27 | -9,27 | Cnt |
| 9 | -2,87 | -29,85 | -17,28 | Cnt |
| 10 | -1,72 | -19,92 | -11,25 | Cnt |
| 11 | -10,77 | -31,94 | -27,18 | Cnt |
| 12 | -2,82 | -34,26 | -21,01 | Cnt |
| 13 | -21,64 | -2,37 | -9,08 | Npls |
| 14 | -18,00 | -2,74 | -8,34 | Npls |
| 15 | -27,16 | -4,41 | -20,27 | Npls |
| 16 | -30,66 | -5,19 | -18,70 | Npls |
| 17 | -22,24 | -3,72 | -6,58 | Npls |
| 18 | -31,05 | -6,17 | -8,50 | Npls |
| 19 | -21,23 | -2,50 | -8,50 | Npls |
| 20 | -25,82 | -2,17 | -13,42 | Npls |
| 21 | -13,38 | -3,54 | -7,03 | Npls |
| 22 | -27,26 | -2,90 | -17,12 | Npls |
| 23 | -25,58 | -7,15 | -4,60 | IFpls |
| 24 | -22,01 | -7,08 | -3,63 | IFpls |
| 25 | -20,61 | -10,47 | -3,69 | IFpls |
| 26 | -18,22 | -16,17 | -4,89 | IFpls |
| 27 | -8,86 | -14,33 | -3,59 | IFpls |
| 28 | -12,24 | -12,37 | -2,03 | IFpls |
| 29 | -25,82 | -22,59 | -10,72 | IFpls |
| 30 | -17,07 | -16,76 | -3,37 | IFpls |
| 31 | -9,91 | -12,66 | -7,28 | IFpls |
| 32 | -11,29 | -11,33 | -7,16 | IFpls |
| 33 | -16,52 | -12,48 | -4,01 | IFpls |
| 34 | -15,16 | -14,65 | -4,91 | IFpls |

**Table S7** LDA confusion matrix for liver samples in spectral region in nucleic acids and polysaccharides (1200-650 cm^-1^). Cnt (control), IFpls (the group receiving plasma from rats undergoing intermittent fasting), and Npls (the group receiving plasma from untreated rats).

| **Confusion matrix** | **Actual** | **Cnt** | **Npls** | **IFpls** |
| --- | --- | --- | --- | --- |
| Predicted |  | 1 | 2 | 3 |
| **Cnt** | 1 | **12** | 0 | 0 |
| **Npls** | 2 | 0 | **10** | 0 |
| **IFpls** | 3 | 0 | 0 | **12** |

**Table S8** LDA confusion matrix for liver samples in spectral region in nucleic acids and polysaccharides (1200-650 cm^-1^). Cnt (control), IFpls (the group receiving plasma from rats undergoing intermittent fasting), and Npls (the group receiving plasma from untreated rats).

|  | **Cnt** | **Npls** | **IFpls** | **Predicted** |
| --- | --- | --- | --- | --- |
|  | 1 | 2 | 3 | 4 |
| 1 | -7,70 | -23,41 | -7,70 | Cnt |
| 2 | -2,87 | -27,81 | -4,98 | Cnt |
| 3 | -5,89 | -23,36 | -6,57 | Cnt |
| 4 | -4,23 | -24,32 | -5,63 | Cnt |
| 5 | -3,71 | -40,22 | -8,59 | Cnt |
| 6 | -4,04 | -44,14 | -10,85 | Cnt |
| 7 | -2,61 | -31,35 | -10,63 | Cnt |
| 8 | -2,68 | -34,60 | -11,37 | Cnt |
| 9 | -2,28 | -40,78 | -10,98 | Cnt |
| 10 | -2,46 | -34,74 | -9,26 | Cnt |
| 11 | -9,73 | -52,75 | -24,40 | Cnt |
| 12 | -2,97 | -43,00 | -14,36 | Cnt |
| 13 | -28,13 | -3,09 | -14,20 | Npls |
| 14 | -37,93 | -3,42 | -23,24 | Npls |
| 15 | -35,30 | -5,64 | -24,17 | Npls |
| 16 | -41,04 | -5,69 | -26,77 | Npls |
| 17 | -34,11 | -4,70 | -16,94 | Npls |
| 18 | -29,25 | -5,11 | -15,99 | Npls |
| 19 | -34,24 | -2,51 | -19,59 | Npls |
| 20 | -36,74 | -3,27 | -20,93 | Npls |
| 21 | -39,81 | -10,51 | -32,88 | Npls |
| 22 | -40,99 | -5,86 | -28,84 | Npls |
| 23 | -10,71 | -9,31 | -4,28 | IFpls |
| 24 | -6,67 | -19,80 | -1,83 | IFpls |
| 25 | -11,60 | -15,08 | -3,31 | IFpls |
| 26 | -17,65 | -13,34 | -6,68 | IFpls |
| 27 | -11,50 | -21,47 | -2,94 | IFpls |
| 28 | -7,39 | -29,47 | -2,66 | IFpls |
| 29 | -4,18 | -23,96 | -3,61 | IFpls |
| 30 | -8,33 | -20,48 | -2,19 | IFpls |
| 31 | -12,39 | -24,03 | -3,12 | IFpls |
| 32 | -12,96 | -31,98 | -7,56 | IFpls |
| 33 | -7,51 | -26,89 | -3,11 | IFpls |
| 34 | -8,12 | -17,56 | -3,56 | IFpls |
